# The value of using a brain laser interstitial thermal therapy (LITT) system in patients presenting with high grade gliomas where maximal safe resection may not be feasible

**DOI:** 10.1186/s12962-016-0055-2

**Published:** 2016-03-21

**Authors:** Jeffrey D. Voigt, Gene Barnett

**Affiliations:** The Rose Ella Burkhardt Chair in Neurosurgical Oncology, The Cleveland Clinic, Lerner College of Medicine of Case Western Reserve University, The Cleveland Clinic S73, 9500 Euclid Avenue, Cleveland, OH 44195 USA; Department of Neurological Surgery, Rose Ella Burkhardt Brain Tumor and Neuro-Oncology Center Cleveland Clinic Neurological Institute, The Cleveland Clinic, S73, 9500 Euclid Avenue, Cleveland, OH 44195 USA; 99 Glenwood Rd, Ridgewood, NJ 07450 USA

**Keywords:** Brain LITT, Cost effectiveness, Value, LYG, Survival

## Abstract

**Background:**

The objective of this analysis was to determine the value (incremental cost/increment benefit) of a brain LITT system versus employing current surgical options recommended by NCCN guidelines, specifically open resection (i.e. craniotomy) methods or biopsy (collectively termed CURRENT TREATMENTS) in patients where maximal safe resection may not be feasible. As has been demonstrated in the literature, extent of resection/ablation with minimal complications are independently related to overall survival.

**Methods:**

A cost effectiveness analysis from a societal perspective was employed using TreeAge Pro 2014 software. Direct costs (using national average Medicare reimbursement amounts), outcomes (overall survival), and value [defined as increment cost/incremental survival—evaluated as cost/life year gained (LYG)] were evaluated. Sensitivity analysis was also performed to determine which variables had the largest effect on incremental costs and outcomes.

**Results:**

In the base case, the overall survival was improved with brain LITT versus CURRENT TREATMENTS by 3.07 months at an additional cost of $7508 (or $29,340/LYG). This amount was significantly less than the current international threshold value for $32,575/LYG and considerably less than the US threshold value of $50,000/LYG. This incremental cost may also qualify under NICE criteria for end of life therapies. In sensitivity analysis: As percent local recurrence GBM increased; cost of DRG25/26 increased; percent GTR increased; and gliadel use increased—the value of brain LITT improved. Additionally, in those patients where a biopsy is the only option, brain LITT extended life by 7 months.

**Conclusions:**

Brain LITT should be considered a viable option for treatment of high grade gliomas as it improves survival at a cost which appears to be of good value to society. This incremental cost is less than the international and US thresholds for good value.

## Background

According the Central Brain Tumor Registry of the US (CBTRUS), there are over 138,000 people in the US living with primary brain and central nervous system malignant tumors primary in the United States [1]. The incidence of primary malignant brain tumors is expected to be over 23,000 in 2015 [1]. Of these types of tumors, >50 % [2–4] (approximately 11,500) are classified as being at high risk [for complications] for resection. This is mainly due to the grade of tumor and these tumors residing in or near areas of eloquence or being deep seated in nature (e.g. tumor residing in the brain stem [also referred to as complex anatomy]). Extent of resection (EOR) with the aim of maximal cytoreduction of the tumor is strongly correlated with outcomes (i.e. survival and function as classified under the Karnofsky performance scale) [5] and the effectiveness of other treatment modalities such as radiation or chemotherapy [6, 7]. One of the main issues with tumors that are in or near areas of eloquence or, that are deep seated in nature, is an inability for neurosurgeons to adequately resect the tumor without causing longer term neurological complications from surgery (i.e. open resection or biopsy or CURRENT TREATMENTS). Craniotomy procedures that have been performed on high-grade gliomas in or near areas of eloquence have historically resulted in neurological complications (i.e. functional and/or cognitive deficits on a neurological basis) that are permanent in nature and result in suboptimal resection. These major complication rates range from 4.5 to 13 % in large cohorts of patients [8–17] and also result in suboptimal EOR of 78–<95 % [5, 8, 12–14]^.^ Further, these acquired neurological complications resulting from surgery result in decreased median survival rates [18]. While the National Comprehensive Cancer Network (NCCN) includes the use of craniotomy or biopsy [19] [referred to as CURRENT TREATMENTS moving forward] for subtotal resection in its treatment algorithms for primary or recurrent glioblastoma (where maximal resection is not safe or feasible), it does not make evidentiary or consensus recommendations on their use [19].

Recently new MRI guided laser interstitial thermal therapy (LITT) systems for ablating neurological soft-tissue have been FDA cleared and; covered and paid for by Medicare (via a new technology add on payment) in treating primary and recurrent gliomas [20, 21]. These technologies, have been reported on extensively in the literature, including in two systematic reviews [22, 23]. These technologies apply focused laser energy which a surgeon uses to ablate tissue such as tumors from the inside of the brain (using a bur or twist drill hole for brain access). Real time MRI thermometry is also used so that surrounding healthy tissue damage can be minimized. These systems allow surgeons to selectively ablate tumors and lesions in the brain that may have been previously deemed inoperable, difficult to access, or unsafe to resect based on their location in or near areas of eloquence.

In examining the use of brain LITT in the peer review literature in the subset of patients whose high grade glioma resides in or near areas of eloquence or; that are deep seated in nature, it was found that the major complication rates directly resulting from the brain LITT surgery were in the 0–6 % range; average of 2.7 % (Table 1), which is lower than 4.5–13 % seen in CURRENT TREATMENTS (Table 2). (Note: The analysis as found in Table 1 shows all types of brain LITT used in high-grade gliomas located in areas of eloquence over the years, with and without the use of MRI guidance.) As well, it has been found that physicians who are experienced in using brain LITT technology in complex anatomy experience an EOR approaching 98 % [24] and that those who are treated with brain LITT experience a length of stay (LOS) that averages 3 days [24]. This is in contrast to patients who are treated with open craniotomy with ICD9CM Diagnosis codes 191.0–191.8, whose LOS average 6.55 ± 1.77 days under diagnostic related groups [DRG] 25–27 (craniotomy with and without comorbidity/complication) [25].

**Table 1:** Studies examining the use of LITT with high grade gliomas in areas of eloquence

Study	Number patients identified with tumors in areas of eloquence	Tumor type	Length of stay	Extent resection	KPS (pre/post)	Major complications (%)^a^
Sakai [42]	3	Denovo glioma	N/A	100 %	Two patients had a 90 and 100 KPS pre-surgery; other not mentioned	0
Reimer [43]	4	Recurrent glioma	Shorter with LITT vs. craniotomy	N/A	N/A	0
Schwarzmaier [40]	16	rGBM	Shorter with LITT vs. craniotomy	N/A	N/A	0
Carpentier [44]	4	rGBM	Patients discharged the next day	100 %	Unchanged pre and post-surgery	0
Jethwa [45]	3	GBM	Median 1 day	100 %	N/A	0
Sloan [46]	8	rGBM	3.75 ± 1.83 days (mean ± SD)	78 ± 12 %	Pre-surgery 85; post 83	0
Schroeder [47]	2	Anaplastic astrocytoma	N/A	92.8 % (mean) [range 77.7–100 %]	Pre-surgery 80	0
Mohammadi [24]	35	rGBM = 19 Glioma/GBM = 16	Median 3 days (range 1–29 days)	98.2 % (median)	Pre-surgery 80	6
Totals	75					(2/75 = 2.7)

*KPS* Karnofsky performance scale, *rGBM* recurrent glioblastoma multiforme, *GBM * glioblastoma multiforme

^a^Major complications = Neurocognitive complications extending >3 months post surgery

**Table 2:** Studies examining the use of craniotomy with high grade gliomas in areas of eloquence

Study	Number patients identified with tumors in areas of eloquence	Tumor type	Length of stay	Extent resection	KPS (presurgery/postsurgery)	Major complications^a^
Sawaya [14]	154	Metastatic disease 48 %; GBM 27 %	Median 5 days	37 % had <95 % EOR	Post 32 % improved; 58 % no change; 9 % deterioration	13 %
Lacroix [13]	79	rGBM 17 % GBM 83 %	N/A	78 %	N/A	9 %
Jackson [16]	78	GBM	N/A	Median 96 %	N/A	12.8 %
Kim [17]	200	Primarily GBM	N/A	125/200 patients had a GTR (63 %) [defined as ≥95 %]; 20/200 (14 %) STR, 46/200 (23 %) partial resection	N/A	11 %
Sanai [9]	40	Insular gliomas WHO Type III & IV	N/A	87.5 % had <90 % EOR	N/A	4.8 %
Kuhnt [12]	79	GBM rGBM	N/A	78.2 %	N/A	9 %
Kreig [11]	47	GBM; anaplastic astrocytoma, diffuse astrocytoma	N/A		90 % going to 80 %	8.5 %
Chaichana [10]	146	GBM	Median 4 days	81 ± 1.6 %	N/A	7.3 % overall. However this was not broken out by complications in areas of eloquence
Schucht [8]	67	GBM	N/A	73 % GTR 27 % STR	N/A	4.5 % with persistent motor deficit

*KPS* Karnofsky performance scale, *GTR* gross total resection, *STR* subtotal resection

^a^Major complications = neurocognitive complications extending >3 months post surgery

In establishing the value of a new treatment, the new option is compared to the weighted costs and outcomes of the combined existing treatments [CURRENT TREATMENTS] for the same patient population. In the United States cost effectiveness ratios of <$50,000/life year gained [LYG] are considered attractive [26]. From an international perspective, <30,000€ (or $32,575 in current US dollars)/LYG [or at $2714/month survival gained] is considered a good value [27].

It is with these facts in mind that an analysis was undertaken to evaluate the direct costs and overall survival of treating complex high-grade gliomas utilizing either brain LITT or CURRENT TREATMENTS (per the NCCN CNS practice guidelines [19]). The costs and overall survival (OS) were evaluated via a cost effectiveness analysis (including sensitivity analysis) as described below. The hypothesis being tested is that the use of brain LITT in these types of patients would be considered cost effective (i.e. of value) at a willingness to pay (WTP of <$32,575/LYG or incremental cost $2714/incremental month of survival) in patients with complex brain anatomy (which included brain tumors in or near areas of eloquence or; in deep seated tumors which are difficult to access via surgery). This analysis examines this incremental cost/incremental survival benefit (termed ICER).

## Methods

A decision tree was developed to evaluate the cost-effectiveness of using brain LITT versus CURRENT TREATMENTS (collectively craniotomy ± gliadel wafer, plus biopsy) in patients with complex anatomy. Additionally, brain LITT was compared to the separate procedures of craniotomy without gliadel wafer, craniotomy w/gliadel wafer, and biopsy only (which collectively make up CURRENT TREATMENTS) in these patients. The software program used was TreeAge Pro 2014, a decision tree/Markov modeling software program widely used in health care for evaluating cost effectiveness.

The decision tree evaluated the initial procedure and the resultant outcome (i.e. gross total resection [GTR]; subtotal resection [STR])—using probabilities as identified in the peer-reviewed literature and as found in Tables 3, 4. Further it was assumed that patients received adjunct care (e.g. chemotherapy, external beam radiation therapy (EBRT) where appropriate based on the EOR and; as per the NCCN guidelines [19], and evidence-based recommendations [28, 29]. Patients were followed through the treatment decision tree until they died. Gross total resection was defined as an EOR of ≥98 % and a subtotal resection (STR) was classified as less than <98 %. The outcome of the surgery was also evaluated based on the resultant Karnofsky performance scale (KPS). The progression free survival (PFS) of the initial procedure was based off of the EOR as found in the literature and as outlined in Tables 3, 4 below. Progression free survival times and KPS were determinants of when/whether a second procedure was performed. Further, patients whose recurring tumor was local in nature were treated with a second surgery and follow on adjunctive treatment where appropriate, based on the clinical guidelines (e.g. ± gliadel wafer [based on evidence from databases and the literature this occurred 10–30 % of the time] [30, 31], systemic chemotherapy). Patients whose tumor was diffuse in nature were treated with either palliative care or EBRT (external beam radiation therapy) ± chemotherapy, depending upon their KPS. As well, patients whose resultant KPS was <70 after surgery were treated with palliative care for the remainder of their lives. Thus as an example: if a primary procedure under brain LITT resulted in a GTR and the outcome was favorable (e.g. KPS >70 post procedure) (note: the primary procedure would include adjunctive EBRT plus chemotherapy)—the PFS time as identified in the literature was used for determining a second procedure. If the tumor recurred locally, a second brain LITT procedure was performed. If the outcome of the second procedure was favorable (e.g. KPS >70) the patient was treated with follow on therapy (e.g. chemotherapy) and then followed for the remainder of their life. The decision tree for treatment followed the clinical guidelines as found in the NCCN CNS clinical practice guidelines [19]^.^ Neurocognitive complication rates as identified above were also used in the model and affected treatment options and downstream costs such as rehabilitation post procedure.

**Table 3:** Variables used in the model

Name	Description	Comment	Root definition	Low	High
Percent_GTR_Neuroblate	Percent of patients with a GTR deemed to be favorable for survival	Data derived from Mohammadi et al. [24]	0.37	0	1
Percent_open_resection	Percent of patients where open cranitomy was performed for a subtotal resection	Data derived from Laws RE et al. [48]	0.78	0	1
Cost_blended_DRG25_26	Cost of craniotomy procedure for a patient with a high grade glioma—weighted average for DRGs 25–26	CRANIOTOMY and ENDOVASCULAR INTRACRANIAL PROCEDURES W CC or MCC (Nat Aver)—weighted average based on frequency of procedurs in Medicare patients for the 2012 calendar year. HCUPNet source	$22,291	0	25,000
Cost_CPT_00210	National average payment amount Medicare for anesthesia	Assumes surgical procedure to last at least 8 h, 30 min	$1012.50	0	1012.5
Cost_CPT_61799	Stereotactic cranial lesion—complex	Cost of creation of an additional stereotactic lesion (each complex lesion) above and beyond that created by CPT 61798 (assume 3 additional lesions)—each lesion @ $325/lesion (Medicare national average payment rate for 2015	$975	0	1406.76
Cost_CPT_61510	National average payment Medicare for a craniotomy to remove a brain tumor	Craniotomy, trephination, for excision of a brain tumor	$2225	0	2225
Cost_CPT_99144	Cost moederate sedation first 30 min	Cost for moderate sedation of patient first 30 min	$19.30	0	19.3
Cost_CPT_99145	Cost moderate sedation each additional 15 min at a cost of $9.40 for each 15 time increment		$9.40s	0	9.4
Cost_CPT_61798	National average payment amount Medicare for stereotactic radiosurgery	Paid as part of the LITT procedure along with CPT 61510 and CPT 61781	$1408	0	1408
Cost_CPT_61800	National average payment Medicare for headframe placement	2015 national average payment rate for head frame placement used in stereotactic radiosurgery	$165	0	235
Cost_CPT_99233	National average Medicare payment for E&M inpatient—subsequent patient visits		$105	0	105
Cost_CPT_99222	National average payment Medicare E&M care first patient visit		$138	0	138
Average_LOS_surgery	Average length of hospital stay in days for patient having surgery in an eloquent area of the brain to remove a brain neoplasm		LOS_surgery_GBM	0	6
Average_LOS_LITT	Average length of hospital stay for patient having LITT surgery for brain tumor		3	0	3
Average_LOS_brain_biopsy	Average lengthe of hospital days for a brain biopsy—subtotal resection	Derived from 2012 HCUPNet inpatient data on ICD9CM 01.13 (closed biopsy brain)	6	0	6
Local_recurrence_GBM	Recurrence GBM that progresses locally	Data derived from Pope WB et al. [49]	0.77	0	1
Cost_Routine_Home_Hospice	Cost for routine home hospice care per day Medicare FY 2015		$159	0	300
Percent_palliative_care	Percent of patients eligible for palliative care based on surviving <6 months	Data derived from Park JK et al. [50]	0.44	0	0.44
Cost_CMG_302	Case mix group 302 for non-traumatic brain injury incurred during neurosurgery procedure. Surgery results in significant motor and cognitive deficits	Assumes patient has “severe” comorbidities and inpatient rehabilitation facility (IRF) is paid at national weighted average of $23,310 for CMG 302 Tier 1 and Tier 2 at the mix of DRG 25 (59 %) and DRG 26 (41 %) (FY 2015 rate for Medicare). Derived from Federal Register, Vol. 79; No. 151, August 6, 2014, page 45888	$23,310	0	20,000
Cost_CPT_99220	Observation care facility setting—initial visit; 2015 Medicare national average payment	Dervied from Medicare PPS FY 2015	$188	0	188
Cost_CPT_99226	Observation care facility setting—subsequent visit; 2015 Medicare national average payment	Dervied from Medicare PPS FY 2015	$106	0	106
Cost_CPT_97001	National average payment Medicare for physical therapy initial evaluation	Medicare PPS FY2015	$75.44	0	75.44
Cost_CPT_97112	National average payment rate for neuromuscular rehabilitation per day	Medicare PFS FY 2015	$33.61	0	33.61
Average_LOS_IRF	Length of stay inpatient rehabilitation facility post complication resulting for neurosurgery in an eloquent area of the brain	Assumes and average LOS of 10 days—derived from Case Mix Group 301 for nontraumatic brain injury from surgery	10	0	10
Cost_APC_0067	CMS payment for APC 0067—cranial SRS	CMS FY 2015 payment rate for aPC 0067 @ $9765.40—for SRS brain	$9765.40	0	20,000
Cost_CPT_77372	National average payment for Medicare for treatment of brain cancer using SRS linear accelerator	Medicare FY 2015 payment rate for SRS linear based	$1063	0	1063
OS_Survival_KPS_70_or_lower	Overall median survival in months with a Karnofsky Performance Score of less than or equal to 70	Derived from Simpson JR et al. [51]	7.8	0	7.8
OS_Survival_KPS_80_or_greater	Overall median survival in months with a Karnofsky Score of 80 or greater	Derived from Lacroix et al. [13]	11.2	0	11.2
OS_survival_SRS	Median survival time in months for patients with SRS after failed surgery	Derived from Niranjan A, et al. [52]	9.03	0	9.03
Cost_CPT_77262	Therapeutic radiation treatment planning—intermediate	Radiation treatment planning for SRS (EBRT)—Medicare reimbursement for CY 2015	$113	0	113
Cost_CPT_77285	Therapeutic radiology simulation—intermediate	Cost for simulation treatment—therapeutic radiology—Medicare CY 2015 payment rate	$428	0	428
Cost_CPT_77306	Cost EBRT isodose plans for radiation therapy	Cost for EBRT isodose planning—Medicare CY 2015	$146	0	146
Cost_CPT_77300	Cost for dosimetry calculations	Medicare reimbursement for dosimetry calculations—CY 2015	$63	0	63
Cost_CPT_77333	Cost for design and construction for treatment devices used to protect normal tissues	Medicare reimbursement CY 2015 for design and construction of treatment devices used to protect normal tissues	$53	0	53
Cost_CPT_77336	Cost for medical physicists time in treatment and planning	Medicare reimbursement for medical physicist CY 2015	$77	0	77
Cost_CPT_77370	Cost medical physicist consultative report	Medicare payment for medical physicist consultative report	$117	0	117
Cost_CPT_77432	Cost reporting patient’s care—review films, review dosimetry and chart	Medicare reimbursement for reporting patient’s care during EBRT—CY 2015	$419	0	419
Cost_CPT_61517	Medicare reimbursement for placement of carmustine wafer placement in patient with high grade glioma	Medicare national average reimbursement amount for placement of carmustine wafer placement in patient with a high grade glioma—2015	$94	0	94
Cost_DRG_23	National average reimbursement amount for DRG 23—craniotomy with major device implantation	National average payment amount for DRG 23—craniotomy with major device implantation—chemo implant (e.g. carmustine wafer. Based on level II evidence, carmustine wafers are recommended in patients for whom craniotomy is indicated. Fadul CE et al. [28]	$31,090	0	31,090
Costs_Planning_EBRT	Combined physician service costs for EBRT	Medicare 2015 reimbursement for planning and reporting on EBRT	Cost_CPT_77262 + Cost_CPT_77285 + Cost_CPT_77300 + Cost_CPT_77306 + Cost_CPT_77333 + Cost_CPT_77336 + Cost_CPT_77370 + Cost_CPT_77432	0	1416
Cost_TMZ_with_radiation	Cost TMZ oral therapy during period of radiation—total of 6 weeks	Cost of TMZ as per Medicare fee schedule J8700 = $9.75/pill. Obtained from 2013 Ingenix HCPCS level II code national average Medicare payment amount. Therefore $9.75 X 42 = $409.50	$410	0	410
Cost_TMZ_per_pill	Cost of TMZ per pill—HCPCS code J8700	Cost of TMZ per pill based on 2013 HCPCS level II code book for J8700 @ $9.75/pill	$9.75	0	9.75
Percent_Gliadel_wafer	Percent of time gliadel wafer used as adjuncitve therapy in high grade glioma post craniotomy	Derived from Price et al. [30]	Percent_Gliadel_wafer_implantations	0	1
Percent_neuro_deficit_Gliadel	Percent of patients experiencing neurological deficit with Gliadel wafer	Derived from Bregy et al. [37]	0.153	0	0
Percent_major_comps_surg_plus_Gliadel	Combination of major complications resulting from major surgery plus Gliadel usage		Percent_neuro_deficit_Gliadel + Percent_major_complications_surgery	0	0
Percent_biopsy_disch_SNF_IRF	Percent of patients with biopsy procedure discharged to SNF or IRF post biopsy	Derived from Malone et al. 2015 World Neursurg—24.4 % of all patients with a stereotactic biopsy discharged either to SNF or IRF	0.244	0	0
Cost_SNF	Cost skilled nursing facility in caring for a patient post brain surgery	Derived from 2015 Medicare rates in caring for a person post brain surgery in a skilled nursing facility	$4930	0	0

**Table 4:** Distributions used in the model

Type	Name	Description	Param 1	Param 2	Param 3
Triangular	Percent_major_complications_surgery	Percent of patients experiencing a major complication from surgergy	0.045	0.09	0.13
Triangular	Percent_major_complications_LITT	Percent of major complications resulting from LITT procedure	0.0	0.027	0.06
Uniform	Percent_GTR_biopsy	Percentage of patients who have a GTR with biopsy	0.0	0.45	
Uniform	Percent_major_complications_biopsy	Percent of major complications resulting from stereotactic biopsy	0.031	0.064	
Uniform	Percent_unresectable_rGBM_surgery	Percent of recurrent GBMS that are unresectable with open craniotomy surgery	0.23	0.38	
Uniform	Percent_open_resection_total	Percent of eloquent areas of brain where a total resection was achieveable	0	0.23	
Uniform	OS_EOR_biopsy	Overall survival in months with biopsy based on an assumed extent of resection of <=70 %	4.85	12	
Triangular	OS_EOR_STR	Overall survival in months with Subtotal Resection biopsy or craniotomy based on an assumed extent of resection of >=85 %	9.7	10.9	12.2
Triangular	OS_EOR_GTR	Overall survival in months with Subtotal Resection biopsy or craniotomy based on an assumed extent of resection of >=98 %	11.4	13.1	14.6
Uniform	Added_Survival_SRS	Additional overall survival in months with use of SRS in patients who have a KPS >=70	7.5	8.5	
Uniform	OS_recurrent_Diffuse_GBM	Overall survival of recurrent diffuse GBM	6	7	
Normal	PFS_LITT_GTR	Progession free survival using LITT—assuming GTR	12.2	13.6	
Normal	PFS_LITT_STR	Progression free survival using LITT with subtotal resection	7.4	7	
Normal	PFS_biopsy_GTR	Progession free survival using biopsy with GTR	12.2	13.6	
Normal	PFS_biopsy	Progression free survival using biopsy with inadequate resection	4.8	5.2	
Normal	PFS_surgery_GTR	Progression free survival with surgery and GTR	12.2	13.6	
Normal	PFS_surgery_STR	Progression free survival with surgery with STR	7.4	7	
Normal	LOS_surgery_GBM	Average LOS and std dev for GBM procedures under DGR 26; 2012 data	6.55	1.77	
Uniform	Timing_follow_on_chemo_TMZ	Amount of time in days of follow on TMZ chemotherapy	180	360	
Normal	Incremental_survival	Incremental overall survival with implanting a carmustine wafer versus not	3.3	4.467	
Triangular	Percent_neuro_comps_0_to_3_mths_surgery	Percent of neurological complications (motor and cognitive lasting 0–3 months requiring rehabilitation; independent of major complications =>4 months	0.06	0.068	0.153
Uniform	Percent_neuro_comps_0_to_3_mths_LITT	Percent of neurological complicaitons (motor and cognitive lasting 0–3 months requiring rehabilitation; independent of major complications =>4 months	0	0.02	
Uniform	Percent_Gliadel_wafer_implantations	Percent of time a Gliadel wafer implanted in a patient for treating brain cancer	0.1	0.33	

**Fig. 1:**
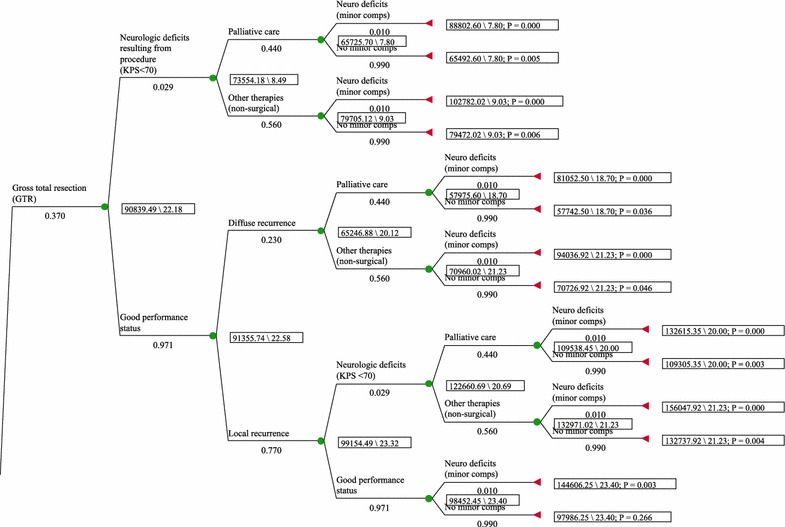
Brain LITT arm of decision tree examining costs/outcomes of patients with a gross total resection

Direct societal costs used in the model were derived from 2015 Medicare national averages for surgery (including hospital in-patient and physician services rendered during the inpatient stay) and follow on care (e.g. radiation therapy, chemotherapy, rehabilitation [for a complication] or palliative/home hospice care). These costs can be found in Tables 3, 4. Table 5 shows what the overall costs for an acute inpatient stay for tumor removal and; is be based on a weighted average use and cost for DRG’s 25–26 (using 2012 Medicare data on the incidence of each procedure and 2015 National average reimbursement rates for Medicare) [32] plus the physician services rendered for each treatment type (brain LITT, craniotomy, biopsy). Table 5 also shows the national average weighted costs for DRG 23 (when craniotomy with gliadel wafer placement was performed). Effectiveness was evaluated as overall survival (OS) of the patient. These values were derived from the literature and based on the outcome of the first and second (if indicated and based on the clinical guidelines) surgical procedures. Costs and OS were discounted at 3 % annually—which is the most commonly used discount rate for medical therapies [33]. Costs and OS used normal distributions along with confidence intervals and standard deviations for probabilistic analysis. Cost effectiveness analysis (incremental cost of using brain LITT plus other interventions (includes such interventions such as: adjunctive therapies, treatment for complications, hospice care) over the life of the patient/LYG; termed incremental cost effectiveness ratio (ICER) moving forward) was analyzed to determine whether the incremental cost/incremental survival was under internationally accepted cost/LYG thresholds. Sensitivity threshold analysis was performed to determine which variables had the greatest effect on the ICER. Lastly, Monte Carlo simulation (expected value for 10,000 simulated trials) was also run. These analyses were performed using TreeAge Pro 2014 (TreeAge Software, Inc., Williamstown, MA). Figure 1 depicts a section of the decision tree related to treatment with brain LITT for gross total resection (≥98 % tumor ablated).

**Table 5:** Costs of care based on Medicare reimbursement to the hospital and physician for brain LITT, craniotomy w/carmustine, craniotomy w/o carmustine, or biopsy inpatient procedures—tumor resection

Cost item	Brain LITT LOS = 3	Craniotomy w/carumstine wafer LOS = 7.5	Craniotomy LOS = 7.5	Biopsy LOS = 6
DRG 25-26—craniotomy	$22,291	–	$22,291	$22,291
DRG 23—craniotomy with chemo implant	–	$31,090	–	–
CPT 00210—anesthesia	$1010	$1010	$1010	$1010
CPT 99144/5—physician observation of sedation	$320	$320	$320	$320
CPT 61510—craniotomy	–	$2225	$2225	–
CPT 61517	–	$94	–	–
CPT 61751		–		$1405
CPT 61781	$235	–		–
CPT 61798	$1410	–		–
CPT 61799	$975	–	–	–
CPT 61800	$165	–		$165
CPT 99222	$138	$138	$138	$138
CPT 99233 @ $105/day	$315	$840	$840	$630
Total	$26,859	$35,717	$26,824	$25,959

*LOS* length of stay in days, *DRG* diagnostic related group, *CPT* current procedural terminology

## Results and discussion

Table 6 compares the overall costs and survival in employing either brain LITT or CURRENT TREATMENTS (again which is comprised of either open resection ± gliadel wafer or biopsy) in cases where high-grade gliomas reside in or near eloquent areas of the brain or are deep seated. As can be seen in the base case, the additional costs (over the lifetime of the patient) with LITT vs. CURRENT TREATMENTS is $7508 and the overall improved survival with brain LITT vs. CURRENT TREATMENTS is 3.07 months. More specifically, as it relates to cost effectiveness, for every month in survival gained, it would cost an additional $2445 in using brain LITT versus CURRENT TREATMENTS. This translates into an incremental cost per LYG of $29,340 when using brain LITT. If one examines brain LITT compared to each option separately (that is the procedures contained within CURRENT TREATMENTS) the increment costs per LYG are: $8458/LYG compared to craniotomy (which consisted of a combination of craniotomy with and without gliadel wafer) and $48,552/LYG when compared to biopsy (Table 7). Table 8 shows similar findings with a Monte Carlo simulation.

**Table 6:** Base case comparing LITT versus OTHER PROCEDURE on the outcomes of costs and overall survival

Treatment	Cost	Overall survival in months
Brain LITT	$89,839	19.04
OTHER TREATMENTS	$82,331	15.97

**Table 7:** Incremental cost/LYG

Therapy	Cost	Overall survival (OS) [months]	Incremental cost/increment month survival in using brain LITT	Incremental cost/LYG using brain LITT
Biopsy	$63,458	12.52	$4046/mth	$48,552/LYG
Craniotomy (includes ± carmustine wafer)	$87,654	16.94	$795/mth	$8458/LYG
OTHER TREATMENTS-combines craniotomy plus biopsy)	$82,331	15.97	$2445/mth	$29,340/LYG
Brain LITT	$89,839	19.04	N/A	N/A

**Table 8:** Monte Carlo simulation (run 10,000 times) comparing LITT versus OTHER PROCEDURE on the outcomes of costs and overall survival

Treatment	Cost	Overall survival in months
Brain LITT	$89,785 ± $15,885	19.12 ± 3.51
OTHER TREATMENTS	$82,042 ± $22,070	15.95 ± 4.04

**Fig. 2:**
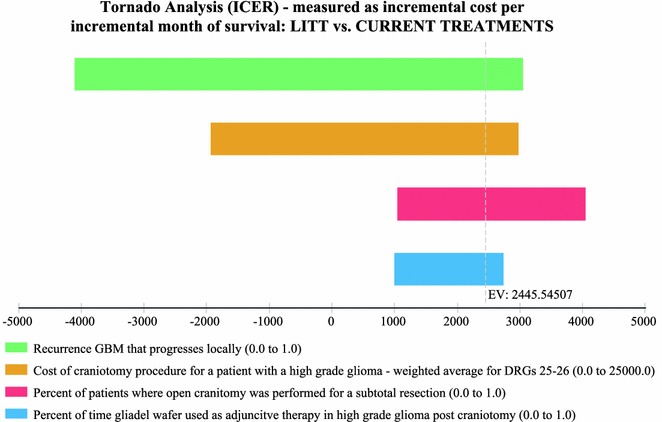
Tornado sensitivity analysis—ICER measured as incremental cost per incremental month survival: LITT versus CURRENT TREATMENTS

**Fig. 3:**
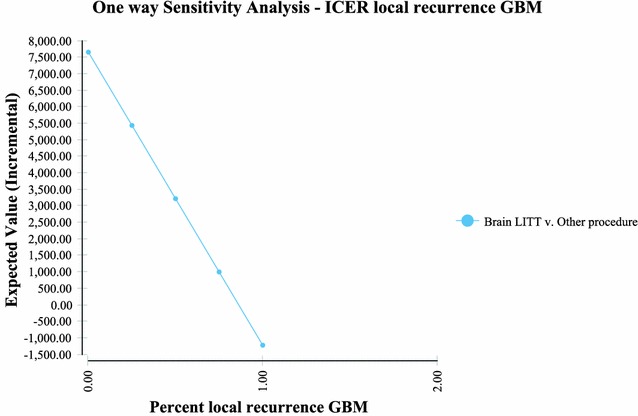
One way sensitivity analysis—ICER local recurrence GBM

**Fig. 4:**
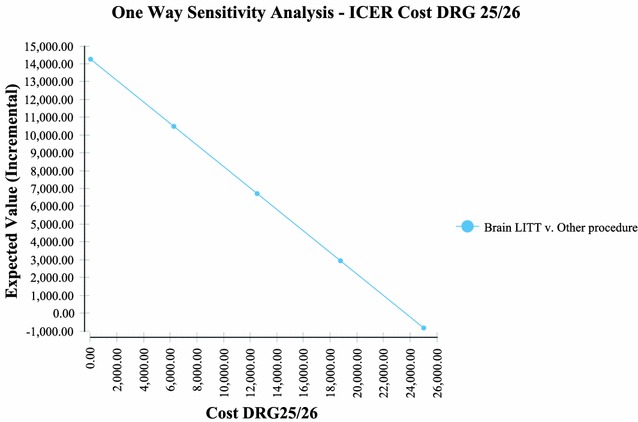
One way sensitivity analysis—ICER DRG 25/26

**Fig. 5:**
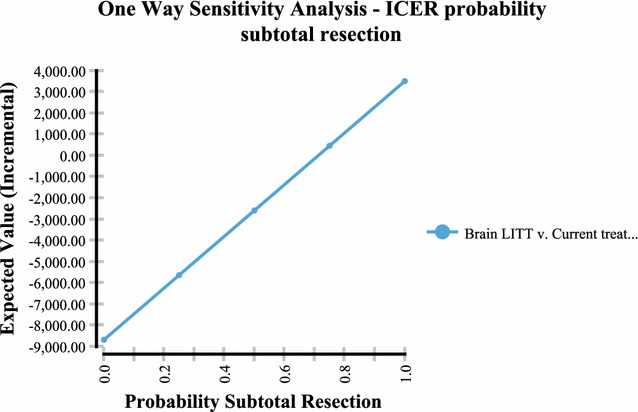
One way sensitivity analysis—ICER percent subtotal resection

**Fig. 6:**
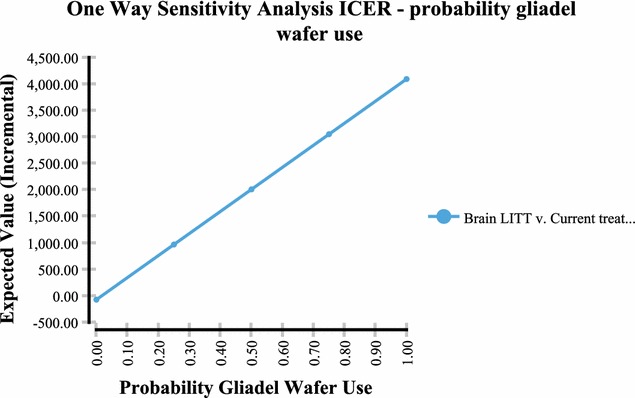
One way sensitivity analysis—ICER percent gliadel wafer use

**Fig. 7:**
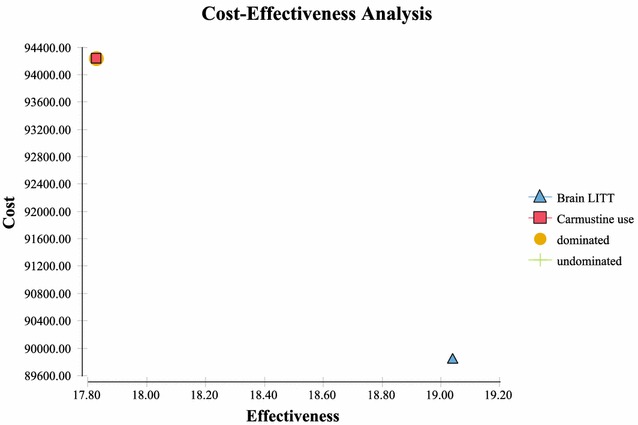
Cost-effectiveness analysis—LITT dominance

Sensitivity analysis performed via a tornado plot (Fig. 2) showed that with a willingness to pay (WTP) of $2714/month of survival (same as the international threshold of $32,572/LYG) the following variables had the greatest effect on the model: Percent local recurrence of the GBM (Fig. 3)—with the higher the occurrence of local GBM recurrence (vs. diffuse recurrence) the more likely brain LITT was to be cost effective; the higher the cost of a craniotomy procedure (i.e. DRG 25/26)—the more cost effective brain LITT became (Fig. 4); the higher the likelihood (or probability) of a subtotal resection (versus GTR); the less cost effective brain LITT became (Fig. 5) and; the higher the probability of use of gliadel wafers, the less cost effective brain LITT became (Fig. 6) (Note that with Fig. 6, the higher use of gliadel wafers resulted in a negative incremental cost/negative OS (resulting in a positive ICER for brain LITT—which in reality shows that the ICER is reflective of the additional cost and additional survival with gliadel versus brain LITT). What Fig. 7 further clarifies is that brain LITT dominates craniotomy plus carmustine use in that it is both less expensive and produces improved overall survival (Note: Strategies that dominate are depicted in the lower right hand corner of cost effectiveness graphs and; strategies that are dominated are shown in the upper left hand corner of the same graph). Lastly Fig. 7 shows that at a WTP of $2714/additional month of survival, the favored strategy is brain LITT (as the WTP intersects with the brain LITT data point).

**Fig. 8:**
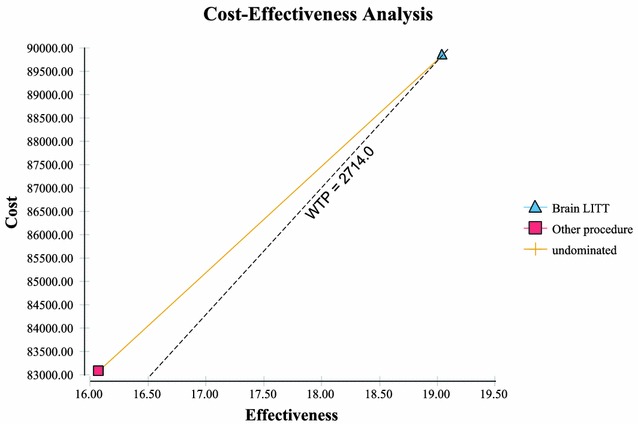
Cost effectiveness analysis—Willingness to Pay

Table 9 shows the likelihoods of a “good performance status (Karnofsky score ≥70) post surgery with and without GTR and; the costs and OS survival associated with each (as calculated by TreeAge 2014). As mentioned above, GTR is defined as an EOR of ≥98 % (with subtotal resection <98 % EOR). What Table 9 also shows is that for the “ideal outcome” for brain cancer surgery (i.e. a GTR with good performance status): this occurs 36 % of the time in brain LITT surgery (with an OS of 22.58 months); 9 % with craniotomy without gliadel wafer (with an OS of 21.75 months) and; 8 % of the time in craniotomy w/gliadel wafer (with an OS of 25.05 months). (NOTE: Biopsy was not factored in this analysis since the result is <98 % EOR). In other words there appears to be 4× higher likelihood of having a good functional outcome along with GTR using brain LITT than with the other options available (Fig. 8).

**Table 9:** Costs and overall survival based on good performance status (Karnofsky ≥70) and extent of resection/ablation—by procedure performed—percent likelihood of event occurring

Procedure	Cost	Overall Survival (months)	% occurrence
Gross total resection/ablation (≥98 % EOR) and good performance status (Karnofsky ≥70)			
Brain LITT	$91,356	22.58	36 %
Craniotomy w/o carmustine wafer	$89,698	21.75	9 %
Craniotomy w/carmustine wafer	$99,013	25.05	8 %
Subtotal resection (STR)/ablation (<98 %) and good performance status (Karnofsky ≥70)			
Brain LITT	$89,721	17.46	61 %
Craniotomy w/o carmustine wafer	$86,493	16.95	49 % (of all OTHER treatments performed)
Craniotomy w/carmustine wafer	$99,679	20.25	15 % (of all OTHER treatments performed)
Biopsy	$62,959	12.72	21 % (of all OTHER treatments performed)

Based on current US (<$50,000/LYG) and International (<30,000€/LYG or $32,575/LYG) threshold values for value, brain LITT should be considered to be “of value” as; its incremental cost/LYG gained (compared to CURRENT TREATMENTS) of $29,340/LYG is less than the thresholds accepted as good value [26, 27]. If one were to examine a comparison in resection of the tumor (i.e. brain LITT compared to craniotomy—the most reasonable side by side comparison to make), the value is significantly improved at $8458/LYG; again an amount considered to be of good value and; well below accepted thresholds internationally and in the US. As well, compared specifically to other cancer therapies, brain LITT represents a better value in money spent in extending survival [27, 34]^.^ Additionally, these findings have important implications for providers, payers, and patients. For providers, the use of brain LITT may extend the overall survival in these types of patients and, at a stable or possibly a better KPS (based on reduced neurological complication rates which in turn would lower KPS scores). Secondly, for payers, this represents good value based on accepted value thresholds. For patients, it appears that OS may be improved based on improved EOR; with less of a likelihood of ending up with surgical complications—which in turn can compromise cognitive and physical functioning.

There are currently no evidence based recommendations for resecting high-grade gliomas when maximal safe resection is not feasible as per the NCCN guidelines [19]. While both subtotal resection and biopsy are mentioned in the NCCN clinical practice guidelines as surgical options, they have significant limitations. Both biopsy and open resection generally result in no or suboptimal resection (<98 %) [5, 8, 12–14], respectively, with a high rate of surgically related complications in the resection group (4.5–13 % [8, 10–14]) when used in these types of patients. As well, patients who had perioperative complications with open resection are less likely to receive radiotherapy or chemotherapy—thus affecting their survivability [27].

The ideal outcome in these types of patients is to achieve GTR without postoperative neurologic complications as; cytoreduction (EOR) without complications plays a very important role in overall survival [12, 27]. Recent advances in less invasive brain LITT (under real time MRI guidance) have produced promising results, with lower complication rates (Table 1). What this decision model demonstrates is that by improving the EOR and lowering procedure related major complications via brain LITT, the overall costs for treating these type of patients over their remaining lives increase minimally (with the additional costs being incurred post procedure via adjunct therapies used to improve OS—based mainly on the ability of clinicians to more frequently/effectively use adjunct therapies with brain LITT). These adjunct therapies can be used due to a greater volume of tumor removal with lower complication rates [6, 7, 35]. The main reason for this is the ability of the brain laser under MRI guidance to selectively ablate cancerous lesions in and around areas of eloquence with less perioperative complications than CURRENT TREATMENTSs. Perioperative complications (including surgically acquired motor, sensory and cognitive deficits) have been found to be an independent risk factor associated with overall survival [35]. An added benefit of the use of brain LITT is a decreased length of hospital stay which in turn also reduces overall costs (i.e. lower physician related costs for inpatient care) [20]. Additionally, a potential benefit of brain LITT versus open resection may be the ability of patients to ambulate more quickly, potentially reducing the incidence of venous thromboembolism (VTE), which can be high in brain cancer and whose risk is further increased post craniotomy [36].

The use of gliadel wafers in high grade gliomas is controversial [37] and their use appears to be practiced judiciously in the US despite being recommended by the NCCN guidelines [19]. The model attempted to account for this and used ranges found in the literature and in publicly available datasets [30, 31].

While the incremental cost/LYG using brain LITT versus biopsy only ($48,552/LYG) exceeds the international threshold of $32,575/LYG, it is lower than the US threshold of <$50,000/LYG and thus would be considered acceptable in the US. Additionally, incremental cost effectiveness ratio (ICER) threshold values established by countries such as the UK (via NICE) for end of life therapies (with the criteria for consideration under this being: life extension of >3 months, small patient population, and prognosis of <24 months) may be more flexible than the threshold set of £20,000–£30,000 for all other therapies/diagnostics/interventions [38]. Thus it may meet the UK threshold. More importantly however, brain LITT should be compared to CURRENT TREATMENTSS as; brain LITT would take place of the other therapeutic options (craniotomy ± gliadel and biopsy) listed under CURRENT TREATMENTS in this patient population. Lastly in this analysis, incremental cost/LYG is likely the more appropriate analysis than incremental cost/quality adjusted life year (QALY) for this condition as; mortality effects are likely to have a more significant impact relative to Quality of Life (QoL analysis); along with the fact that time and resource use are especially constrained in this condition. In these type of circumstances, analysts typically have chosen LYG versus QALY [39].

As it relates to limitations of this analysis the following should be noted:

It was not possible to examine KPS as an outcome based on the small number of patients where this was evaluated. However, and as mentioned above, post-surgery KPS is reflective of neurological complications resulting from surgery. Thus in aggregate, KPS would likely have been higher in the brain LITT arm of the decision tree.

Early studies with brain LITT demonstrate a learning curve and as clinicians gain more experience, it appears that the outcomes improve [24, 40]. Additionally, the outcomes/data used for “CURRENT TREATMENTS (open resection or biopsy)” are well established. It is possible that as time progresses and brain LITT becomes better established (especially for use in these types of patients) that the overall outcomes would improve for brain LITT.

Complications resulting in repeat surgery from chemotherapy implants (e.g. gliadel wafer); craniotomy, brain LITT and biopsy were not evaluated for cost and their effect on overall survival. These complications resulted in approximately a 3 % repeat surgery rate and are highest in the gliadel wafer group [29, 37].

It was assumed based on level II evidence and in the literature reviewed that gliadel wafers were implanted in approximately 10–33 % of patients in the open resection arm of CURRENT TREATMENTS [28, 29]. This may not be the case in all situations. If not used in all instances, the overall costs for the open resection arm (of CURRENT TREATMENTS’s performed) would be less (however to the detriment of overall survival). It was also assumed in the decision tree model that the application of a chemotherapy implant conferred a 3.3 month increment in overall survival [29].

Fluorescent guided surgery using 5-ALA may not be entirely accurate in identifying complete resection and is not FDA-approved in the United States [41]. Some of the studies where EOR was evaluated for craniotomy used this technology [3, 8]. Thus the EOR for craniotomy may have been overestimated—which in turn affected the overall survival numbers in this arm of the decision tree.

Extent of ablation (EOA) from the recent Mohammadi study was used for the brain LITT arm of the trial as a proxy for EOR [24]. In this study the EOA was defined by thermal damage threshold (TDT) lines. Since there is no data in the literature regarding the EOA of thermal damage by brain LITT, it was assumed that the TDT lines defined EOR and, because of this, the 98 % figure was used for EOR in the brain LITT arm of the decision tree model for both PFS and OS. In a prior review of the literature, EOA and EOR, were considered equivalent for PFS [24]. Lastly, since OS had not been followed out long enough for brain LITT, it was also assumed that the 98 % value for EOA with brain LITT assumed a similar OS trajectory as craniotomy.

## Conclusions

The use of brain LITT under MRI guidance in complex craniotomies where high-grade gliomas reside in or near areas of eloquence (or where these types of tumors are deep seated) appears to be cost effective—providing value based on it being lower than “value” thresholds established by policy makers. The implications are that brain LITT should be considered a treatment option in these types of high-risk patients.

## Abbreviations

CNS: central nervous system
DRG: diagnostic related group
EOR: extent of resection
EOA: extent of ablation
GTR: gross total resection
KPS: karnofsky performance scale
LITT: laser interstitial thermal therapy
LOS: length of stay in days
MRI: magnetic resonance imaging
NCCN: national comprehensive cancer network
OS: overall survival
CURRENT TREATMENTS: traditional surgical management as defined by the NCCN guidelines for CNS cancers
PFS: progression free survival
RT: radiation therapy
STR: subtotal resection
WTP: willingness to pay

## References

[1] Central Brain Tumor Registry of the United States (CBTRUS). 2014. http://www.cbtrus.org/reports/reports.html. Accessed 29 Apr 2015.

[2] Chang EF, Smith JS, Change SM, Lamborn KR, Prados MD, Butowski N, Barbaro NM, Parsa AT, Berger MS, McDermott MM (2008). Preoperative prognostic classification system for hemispheric low-grade gliomas in adults. J Neurosurg.

[3] Stummer W, Pichlmeier U, Meinel T, Wiestler OM, Zanella F, Ruelen H-J, ALA-Glioma Study Group (2006). Flourescence-guided surgery with 5-aminolevulinic acid for resection of malignant glioma: a randomized controlled multicentre phase III trial. Lancet Oncol.

[4] Ostrom QT, Gittleman H, Liao P, Rouse C, Chen Y, Dowling J, Wolinsky Y, Krucho C, Barnholtz-Sloan J (2014). CBTRUS statistical report: primary brain and central nervous system tumors diagnosed in the United States in 2007–2011. Neuro-Oncol.

[5] Almenawer SA, Badhiwala JH, Alhazzani W, Greenspoon J, Farrokhyar F, Yarascavitch B, Algird A, Kachur E, Cenic A, Sharieff W, Klurfan P, Gunnarsson T, Ajani O, Reddy K, Singh SK, Murty NK (2015). Biopsy versus partial versus gross total resection in older patients with high-grade glioma: a systematic review and meta-analysis. Neuro-Oncol.

[6] Vuorinen V, Hinkka S, Färkkilä M, Jääskeläinen J (2003). Debulking or biopsy of malignant glioma in elderly people—a randomized study. Acta Neurochir (Wien).

[7] Rubin P, Fischack J, Issacson S (1993). Influence of location and extent of surgical resection on survival of patients with glioblastoma multiforme: results of three consecutive Radiation Therapy Oncology Group (RTOG) clinical trials. Int J Radiat Oncol Biol Phys.

[8] Schucht P, Seidel K, Beck J, Murek M, Jilch A, Wiest R, Fung C, Raabe A (2014). Intraoperative monopolar mapping during 5_ALA-guided resections of glioblastomas adjacent to motor eloquent areas: evaluation of resection rates and neurological outcome. Neurosurg Foc.

[9] Sanai N, Polley M-Y, Berger MS (2010). Insular glioma resection: assessment of patient morbidity, survival and tumor progression. J Neurosurg.

[10] Chaichana KL, Jusue-Torres I, Navarro-Ramirez R, Raza SM, Pascual-Gallego M, Ibrahim A, Hernandez-Hermann M, Gomez L, Ye X, Weingart JD, Olivi A, Blakeley J, Gallia GL, Lim M, Brem H, Quinones-Hinojosa A (2014). Establishing percent resection and residual volume thresholds affecting survival and recurrence for patients with newly diagnosed intracranial glioblastoma. Neuro-Oncol.

[11] Krieg SM, Schnurbus L, Shiban E, Droese D, Obermueller T, Buchman N, Gempt J, Meyer B, Ringel F (2013). Surgery of highly eloquent gliomas primarily assesses as non-resectable: risks and benefits in a cohort study. BMC Cancer.

[12] Kuhnt D, Becker A, Guanslandt O, Bauer M, Buchfelder M, Nimsky C (2011). Correlation of the extent of tumor volume resection and patient survival in surgery of glioblastoma multiforme with high-field intraoperative MRI guidance. Neuro-Oncol.

[13] Lacroix M, Abi-Said D, Fourney DR, Gokaslan ZL, Shi W, DeMonte F, Lang FF, McCuteheon IE, Hassenbusch SK, Holland E, Hess K, Michael C, Miller D, Sawaya R (2001). A multivariate analysis of 416 patients with glioblastoma multiforme: prognosis, extent of resection, and survival. J Neuorsurg.

[14] Sawaya R, Maarouf H, Schoppa D, Schoppa D, Hess KR, Shu W, Wei-Mong S, Wildrick DM (1998). Neurosurgical outcomes in a modern series of 400 craniotomies for treatment of parenchymal tumors. Neurosurg.

[15] Seicean A, Seicean S, Schiltz NK, Alan M, Jones PK, Neuhauser D, Weil RJ (2013). Short-term outcomes of craniotomy for malignant brain tumors in the elderly. Cancer.

[16] Jackson RJ, Fuller GN, Abi-Said D, Lang FF, Gokaslan ZL, Shi WM, Wildrick DM, Sawaya R (2001). Limitations of stereotactic biopsy in the initial management of gliomas. Neuro-Oncol.

[17] Kim SS, McCutcheon IE, Suki D, Weinberg JS, Sawaya R, Lang FF, Ferson D, Heimberger AB, DeMonte F, Prabhu S (2009). Awake craniotomy for brain tumors near eloquent cortex: correlation of intraoperative cortical mapping with neurological outcomes in 309 consecutive patients. Neurosurgery.

[18] McGirt MJ, Mukherjee D, Chaichana KL (2009). Association of surgically acquired motor and language deficits on overall survival after resection of glioblastoma multiforme. Neurosurgery.

[19] NCCN Clinical practice guidelines in oncology. Center nervous system cancers. Version 2.2014. Accessed July 2, 2015 at: NCCN.org.

[20] Federal Register 75 FR 50144, Medicare Program; Hospital Inpatient Prospective Payment Systems for Acute Care Hospitals and the Long-Term Care Hospital Prospective Payment System Changes and FY2011 Rates; Provider Agreements and Supplier Approvals; and Hospital Conditions of Participation for Rehabilitation and Respiratory Care Services; Medicaid Program: Accreditation for Providers of Inpatient Psychiatric Services, August 16, 2010. 20712087

[21] http://www.medtronic.com/for-healthcare-professionals/products-therapies/neurological/laser-ablation/visualase/.. Accessed July 13, 2015.

[22] Voigt JD, Torchia M (2014). Laser interstitial thermal therapy with and without MRI guidance for treatment of brain neoplasms—a systematic review of the literature. Phot Las Med.

[23] Missios S, Bekelis K, Barnett GH (2015). Renaissance of laser interstitial thermal ablation. Neurosurg Focus.

[24] Mohammadi AM, Hawasli AH, Rodriguez A, Schroder JL, Laxton AW, Elson P, Tatter SB, Barnett GH, Leuthardt EC (2014). The role of laser interstitial thermal therapy in enhancing progression free survival of difficult-to-access high-grade gliomas: a multicenter study. Cancer Med.

[25] HCUPNet database query using ICD9CM diagnosis codes 191.0–191.9 (malignant neoplasm of brain). Accessed 30 Apr 2015 at: http://hcupnet.ahrq.gov/.

[26] Cohen DJ, Reynolds MR (2008). Interpreting the results of cost-effectiveness studies. JACC.

[27] Casasdo MA, Benavides M, Cajaraville G, Carreras MJ, Tabernero JM (2007). Cost-effectiveness analysis and budget impact analysis of the first line therapy for metastatic colorectal cancer in Spain. Rev Esp Econ Salud.

[28] Fadul CE, Wen PY, Kim L, Olson JJ (2008). Cytotoxic chemotherapeutic management of newly diagnosed glioblastoma multiforme. J Neurooncol.

[29] Chowdhary SA, Ryken T, Newton HB (2015). Survival outcomes and safety of gliadel wafers in the treatment of high-grade gliomas: a meta-analysis. J Neurooncol.

[30] Price SJ, Whittle IR, Ashkan K, Grundy P, Cruickshank G, UK-HGG Study Group (2012). NICE guidance on the use of gliadel wafers in high grade gliomas: a national study on variation in practice. Brit J Neurosurg.

[31] Data derived from HCUPNet query on 20 July 2105 using procedure codes 00.10 (wafer placement) and 01.51 (excision tumor). http://hcupnet.ahrq.gov/.

[32] http://hcupnet.ahrq.gov/. Accessed June 5 2015.

[33] Gold M, Siegel J, Russell L, Weinstein MC (1996). Cost-effectiveness in health and medicine: report of the panel on cost-effectiveness in health and medicine.

[34] Gil JM, Rubio-Terrés C, Del Castillo A, González P, Canorea F (2006). Pharmacoeconomic analysis of adjuvant therapy with exemsetane, anastrozole, letrozole or tamoxifen in post-menopausal women with operable and estrogen receptor-positive breast cancer. Clin Tansl Oncol.

[35] Orringer D, Lau D, Khatri S, Zamora-Berridi GJ, Zhang K, Wu C, Chaudhary N, Sagher O (2012). Extent of resection in patients with glioblastoma: limiting factors, perception of resectability, and effect on survival. J Neurosurg.

[36] Chandana SR, Movva S, Arora M, Singh T (2008). Primary brain tumors in adults. Am Fam Physician.

[37] Bregy A, Shah AH, Diaz MV, Pierce HE, Ames PL, Diaz D, Komotar RJ (2013). The role of gliadel wafers in the treatment of high-grade gliomas. Expert Rev Anticancer Ther.

[38] NICE. Appraising life-extending end of life treatments. London: National Institute for Health and Clinical Excellence, 2009. p. 1–3.

[39] Chapman RH, Berger M, Weinstein MC, Weeks JC, Goldie S, Neumann PJ (2004). When does quality-adjusting life-years matter in cost-effectiveness analysis. Health Econ.

[40] Schwarzmaier HJ, Eickmeyer F, von Tempelhoff W, Fiedler VU, Neihoff H, Ulrich SD, Yaong Q, Ulrich F (2006). MR-guided laser induced interstitial thermotherapy of recurrent glioblastoma multiforme: preliminary results in 16 patients. Eur J Radiol.

[41] Eyüpoglu IY, Hore N, Savaskan NE (2012). Improving the extent of malignant glioma resection by dual operative visualization approach. PLoS One.

[42] Sakai T, Fujishemia I, Sugiyama K, Ryu H, Uemura K (1992). Interstitial laserthermia in neurosurgery. J Clin Med Surg.

[43] Reimer P, Bremer C, Horch C, Morgenroth C, Allkemper T, Schuierer G (1998). MR-monitored LITT as a palliative concept in patients with high grade gliomas: preliminary clinical experience. J Magn Reson Imag.

[44] Carpentier A, McNichols RJ, Stafford RJ, Itzcovitz J, Guichard JP, Reizine D (2008). Real-time magnetic resonance-guided laser thermal therapy for focal metastatic brain tumors. Neurosurgery.

[45] Jethwa PR, Barrese JC, Gowda A, Shetty A, Danish SF (2012). Magnetic resonance thermotherapy-guided laser-induced thermal therapy for intracranial neoplasms: initial experience. Neurosurgery.

[46] Sloan AE, Ahluwalia MS, Valerio-Pascua J, Manjila S, Torchia MG, Jones SE (2013). Results of the Neuroblate system first-in humans phase 1 clinical trial for recurrent glioblastoma: clinical article. J Neurosurg.

[47] Schroeder JL, Missios S, Barnett GH (2014). Laser interstitial thermal therapy as a novel treatment modality for brain tumors in the thalamus and basal ganglia. Phon Las Med.

[48] Laws RE (2003). Survival following surgery and prognostic factors for recently diagnosed malignant glioma: data from the Glioma Outcomes Project. J Neurosurg.

[49] Pope WB et al. Patterns progression in patients with GBM at first or second relapse treated with Bevacizumab aloe on in combination with irinotecan in the brain study. Neuro Oncol. 2009: 626. **(abstract number 270)**.

[50] Park JK (2010). Scale to predict survival after surgery for recurrent GBM. J Clin Oncol.

[51] Simpson JR (1993). Influence of locaiton and extent of surgical resection on survival of patients with GBM: results of three consecutive radiation therapy oncology group (RTOG) clinical trials. Int J Rad Oncol Biol Phys.

[52] Niranjan A (2015). Role of adjuvant or salvage radiosurgery in the managment of unresected residual or progressive GBM in teh pre-bevacizumab era. J Neurosurg.

